# Development of a Novel Method for Identification of *Alaria alata* Mesocercariae by Matrix-Assisted Laser Desorption/Ionization Time-of-Flight Mass Spectrometry

**DOI:** 10.3390/microorganisms9081664

**Published:** 2021-08-04

**Authors:** Carolyn Kästner, Peter Bahn, Ralph Schönfelder, Zanda Ozoliņa, Laura Alksne, Martin Heinrich Richter, Gunita Deksne, Anne Mayer-Scholl, Annette Johne

**Affiliations:** 1 Department for Biological Safety, German Federal Institute for Risk Assessment (BfR), 12277 Berlin, Germany; carolyn.kaestner@bfr.bund.de (C.K.); peter.bahn@bfr.bund.de (P.B.); martin.richter@bfr.bund.de (M.H.R.); Anne.Mayer-Scholl@bfr.bund.de (A.M.-S.); 2 Food Inspection and Veterinary Department, Administrative District Görlitz, 02708 Löbau, Germany; Ralph.Schoenfelder@kreis-gr.de; 3 Institute of Food Safety, Animal Health and Environment (BIOR), LV-1076 Riga, Latvia; zanda.ozolina@bior.lv (Z.O.); laura.alksne@bior.lv (L.A.); gunita.deksne@bior.lv (G.D.); 4 Faculty of Biology, University of Latvia, LV-1004 Riga, Latvia

**Keywords:** *Alaria alata*, MALDI-TOF MS, diagnostics, detection, identification, trematodes, wild boars, foodborne parasitology

## Abstract

*Alaria* (*A.*) *alata* mesocercariae (AM) have increasingly appeared as incidental findings during the mandatory inspection of wild boars for *Trichinella* in many European countries. An *Alaria* spp.-specific PCR is available for the identification of AM; however, it is time- and cost-intensive. Therefore, we propose a rapid and cost-efficient MALDI-TOF assay for the identification of AM in wild boar meat that can be applied in routine diagnostics. In this study, a fast and methodologically simple protocol for the protein extraction of AM from different host species in different countries was established, and an AM-specific reference spectra database was created as part of the ongoing development of an existing *Trichinella* spp. database. A formic acid protein extraction was performed after pooling 10 AM from the same host individual. In total, 61 main spectra profiles (MSPs) from different host individuals were stored in an AM-specific MSP library. The cluster analysis of these 61 MSPs indicated a possible variation within the *A. alata* species with a tentative association with the geographical origin of the host, but not the host species. This MALDI-TOF assay allows for a fast verification of the AM isolates, which is the next step in the development of a universal database for the identification of several parasites isolated from meat.

## 1. Introduction

To ensure that meat from livestock or game is safe for human consumption, European legislation lays down rules for mandatory *Trichinella* testing such as Commission Implementing Regulation (EU) No. 2015/1375 [1] and subsequent amendments such as Commission Implementing Regulation (EU) No. 2020/1478 [2]. During the inspection of game meat, wide varieties of parasites, which do not belong to the *Trichinella* genus, are frequently detected [3].

One such parasite is *Alaria* (*A.*) *alata*, whose mesocercariae (AM) have been found with increased frequency in wild boars in Europe during the past few years [4,5,6,7,8,9,10,11,12,13].

The adult worms of this parasite live in the intestine of carnivores (e.g., foxes, dogs) and have a complex three-host life cycle that includes wild boars as paratenic hosts [14,15] resulting in possible exposure of humans to the parasite through the consumption of wild boar meat. To date, no human infections caused by the species *A. alata* have been reported. However, Odening [14] demonstrated that primates can function as paratenic hosts for *A. alata*. In addition, *A. alata* was recently classified as a zoonotic parasite of risk group 2 by the Federal Office for the Environment (FOEN) and the Federal Office of Public Health (FOPH) in Bern, Switzerland [16], as well as the Committee on Biological Agents (ABAS) in Germany [17]. However, based on a specific formula, Ozoliņa et al. [10] demonstrated that the probability for humans becoming infected with AM through consumption of wild boar meat ranges between 0.2% and 2.2%.

To assess the risk of human infection with this parasite and better understand regional and spatial fluctuations of AM in wild boars, several studies on the prevalence of *A. alata* in Germany and other European countries were conducted. In Germany, prevalences between 4.7 and 28.3% were described in different regions [4,11,18]. Further studies were reported from France (0.6%) [7], Italy (1.0%) [13], northern Serbia (3%) [19], Austria (6.7%) [20] and Czech Republic (6.8%) [6]. However, significantly higher prevalences were observed in north-eastern Poland (44.3%) [12] and in Latvia (43.9%) [10].

As the artificial digestion technique, which is the gold standard method for *Trichinella* testing, is not sufficiently sensitive for AM detection in meat, Riehn et al. [21] developed the *A. alata* mesocercariae migration technique (AMT) followed by the morphological identification of AM, which, however, requires a professional expertise in parasitology. Therefore, a confirmation of the AMT results using standardized detection methods, such as molecular or protein-based tools, is absolutely essential for the reliable identification of AM. Molecular methods for AM detection include a specific PCR targeting a 303 base pair (bp) sequence within the complete small subunit ribosomal RNA gene (ssrDNA) and the partial (D1-D3) large subunit ribosomal RNA gene (lsrDNA) of the *A*. *alata* genome [22] as well as a 18S rDNA and cytochrome C oxidase subunit I (COI) PCR followed by sequence analysis [23]. However, molecular techniques can be relatively work-intensive, time-consuming and expensive [24].

In recent years, matrix-assisted laser desorption/ionization time-of-flight mass spectrometry (MALDI-TOF MS) has evolved as a routine method for the identification of different microorganisms in many laboratories [25,26,27]. The advantage of the MALDI-TOF technique is that once a robust, generalized protein extraction protocol and a database incorporating a variety of protein spectra from different microorganisms have been established, there is no need to perform a multitude of different assays to identify the pathogen, resulting in fast and cost-effective identification [25]. In routine diagnostics, MALDI-TOF MS has become a standard tool for identification of bacteria and yeast [25,26,27] and has also been applied in parasitology research [28,29,30,31]. However, in the field of foodborne parasitology, only two studies have demonstrated the use of this technique in the framework of official meat inspection for *Trichinella* spp. [24,32].

Therefore, the aim of this study was to establish a rapid, cost-efficient and methodologically simple protocol for protein extraction of AM and to create an AM-specific main spectra profile (MSP) library as an add-on development to an existing *Trichinella* spp. database.

## 2. Materials and Methods

### 2.1. Sample Collection

A total of 61 AM samples from different host individuals were collected during a prevalence study from 2017 to 2020 in Brandenburg, Germany [11], in collaboration with the Food Inspection and Veterinary Department, Administrative District Görlitz (State of Saxony, Germany), the LADR GmbH Medical Care Center North in Flintbek (State of Schleswig-Holstein, Germany), the local veterinary office in Brodnica (Brodnica, Poland) and the Institute of Food Safety, Animal Health and Environment (BIOR) in Riga, Latvia [10,33,34].

All sampled animals were hunted according to each country’s hunting regulations, or other permits if necessary.

During the period from 2017 to 2019, amphibians were collected with special permission (26/2017-E; 06.05.2017, 14/2018-E; 10.05.2018 and 21/2019-E-07.05.2019) that was granted by the Latvian authorities for the collecting and euthanizing of amphibians for scientific purposes (26/2017-E, 14/2018-E, 21/2019-E—Nature Conservation Agency of Latvia).

Adult frogs and tadpoles were gathered from shallow portions using a standard O-frame net with a diameter of 0.6 m, 5-mm mesh size and a handle length of 1.5 m. The collected samples were placed in a disposable box with water (300 mL) and transported to the laboratory within 8 h and kept at +4 °C until further procedures. Euthanasia was performed in the laboratory by a blow to the head as per European Union requirements and the Federation of European Laboratory Animal Science Association regulations (FELASA) [35], under the supervision of a FELASA-certified specialist.

All muscle samples from Saxony, Schleswig-Holstein and Poland were taken from the diaphragm, pharynx or masticatory musculature (Mm. masseter, temporalis, pterygoidei) of wild boars (*Sus scrofa*) that were examined during routine *Trichinella* inspection and transported to the German Federal Institute for Risk Assessment (BfR) in cooling boxes (Table 1). There, the muscle samples were refrigerated at +4 ± 2 °C before being analyzed with the *A. alata* mesocercariae migration technique (AMT) [21] within 24–48 h after arrival in the laboratory.

During the prevalence study in Brandenburg, whole tongue and about 30 g of abdominal fat tissue were collected from wild boars during hunts, refrigerated at +4 ± 2 °C and transported to the BfR within 3 h.

Prior to examination at the BfR using the AMT [21], all samples were refrigerated at +4 ± 2 °C. The average storage time was 24–48 h, but a few samples were kept for up to 7 days when the number of collected samples could not be processed faster.

Viable AM were detected and provisionally identified based on morphological characteristics, considering the number of glandular cells, body shape, size and movement characteristics using the stereomicroscope at 20× to 100× magnification [15].

All collected AM were stored separately for each host individual in ethanol absolute (Fa. Merck, Darmstadt, Germany) at −20 °C.

All AM from the wild boars, European water frogs (*Pelophylax esculentus* complex), common frogs (*Rana temporaria*) and the Eurasian lynx (*Lynx lynx*) from Latvia were isolated from the diaphragm, tongue, head and inner organs. While AMT [21] was used for the detection of AM in wild boars and the Eurasian lynx, the compression method [36,37] was applied for the isolation of AM from European water frogs and common frogs at BIOR in Riga, Latvia for research purposes. These AM were then transported to the BfR in cooling boxes and stored in ethanol absolute at −20 °C after arrival in our laboratory (Table 1).

In addition to the AM samples, three further samples containing *Opisthioglyphe ranae* larvae from European water frogs from Latvia were provided by BIOR and included in this study to verify that the developed MALDI-TOF technique also allows for a reliable differentiation between trematode species. These samples were also transported to the BfR in cooling boxes and stored in ethanol absolute at −20 °C after arrival.

### 2.2. Molecular Species Identification of the Samples

For DNA extraction, the QIAamp^®^ DNA Mini Kit (QIAGEN, Hilden, Germany, 51306) following an adapted Quick-Start Protocol was used. For the elution step, 25 µL of DNA-free water were used and incubated at room temperature for 3 to 5 min before centrifugation at 8000 rpm. This elution step was repeated once. Apart from the elution step, the protocol was performed according to the manufacturer’s instructions.

The DNA extracts from all 61 AM samples were tested by the *Alaria* spp.-specific PCR [22]. All PCR-positive samples were used for the creation of main spectra profiles (MSPs) and stored in the AM-specific MSP library.

Further, the 18S PCR protocol published by Karadjian et al. [3] for the identification of nematodes was applied to 20 randomly selected AM samples, followed by a sequence analysis to determine the suitability of the PCR for the detection and identification of *A. alata*.

In addition, the three samples containing *Opisthioglyphe ranae* larvae were examined using this 18S PCR [3], followed by a sequence analysis, giving a total of 23 larvae-containing samples that were tested by this PCR.

Both the *Alaria* spp.-specific PCR and the 18S PCR were carried out using the protocols published by Riehn et al. [22] and Karadjian et al. [3], respectively.

All PCR products generated by the 18S PCR [3] were sent to Eurofins Genomics (Ebersberg, Germany) for sequence analysis.

### 2.3. Development of a Protein Extraction Protocol for MALDI-TOF Mass Spectrometry

In general, the AM needed to be fixed in alcohol as handling of the motile larvae was neither practical nor repeatable. 

To find a suitable method for protein extraction, several protein sample preparation protocols were tested:

(i) A protocol published by Mayer-Scholl et al. [24] was tested for AM protein extraction but did not result in the generation of reproducible and recognizable protein spectra. 

(ii) A single AM was spotted onto the target, followed by air drying, the addition of 70% formic acid and saturated α-cyano-4-hydroxy-cinnamic acid (HCCA). This protocol did not lead to high-quality protein spectra.

(iii) Finally, we developed a working protocol for protein extraction which allows for the generation of main spectra profiles.

To create MSPs, 10 AM isolated from the same host individual and stored in ethanol absolute at −20 °C were used. These AM were washed three times in 96% ethanol and transferred in 10 µL of 96% ethanol in a 0.2 mL Eppendorf tube. The tube was incubated with an open lid in a thermoblock at 50 °C for approximately 30 min or until the liquid had evaporated. The dried AM were visually controlled by light microscopy. For cell disruption, 10 µL of 70% formic acid were added to the dried larvae and mixed by pipetting slowly up and down at least 20 times. Subsequently, the sample was incubated for 10 min at room temperature before spotting onto the target.

Further, the described protocol was optimized for the use of only one single AM using 3 µL of 70% formic acid for cell disruption. All other steps were performed as described above. For optimization, the AM from the same samples as were used for the protocol based on 10 AM were applied.

To verify that the protein extraction protocol based on one single AM is replicable, a total of 38 samples containing one AM each were examined by the BfR. These AM samples came from five different German wild boars (Saxony (2), Brandenburg (3)) and were included in the AM-specific database.

### 2.4. Generation of an AM-Specific MSP Library

An amount of 1 µL of each protein sample was spotted onto the target plate eight times (MSP 96 target polished steel (MicroScout Target) plate; Bruker Daltonics, Bremen, Germany). For calibration of the MALDI-TOF measurement, 1 µL of Bacterial Test Standard (Bruker Daltonics, Bremen, Germany) was spotted onto the target two times. The air-dried spots were overlaid with 0.8 µL of saturated α-cyano-4-hydroxy-cinnamic acid (HCCA) matrix solution (Bruker Daltonics, Bremen, Germany) and dried completely.

The MALDI-TOF MS measurements were carried out using the MALDI-TOF Microflex LT (Bruker Daltonics, Bremen, Germany) with a range of 2000–20,000 *m/z* (mass to charge ratio). A total of 24 single spectra per protein sample were acquired from eight spots, whereby each spot was measured three times.

For each single spectrum, 200 laser shots in 40 shot steps from different positions of the target spot (random walk motion) were automatically generated using AutoXecute acquisition mode in FlexControl software (Bruker Daltonics, Bremen, Germany), which was slightly adapted in initial laser power (45%) and maximal laser power (65%). The quality of each spectrum was assessed with FlexAnalysis software (Bruker Daltonics, Bremen, Germany). The spectra with peak deviations exceeding 500 ppm were not transferred to the MSP library. All high-quality spectra were added to the database using Biotyper 3 software (Bruker Daltonics, Bremen, Germany).

### 2.5. Evaluation of Generated Protein Spectra by Log Score Values

Log score values are used to measure the reliability of genus and species identification. These values are generated by the comparison of an unknown spectrum with the MSP library as well as the matched peak intensities. The following cutoff scores recommended by Bruker were utilized for sample identification: 0 to 1.699 indicates “no reliable identification”; 1.7 to 1.999 means “probable genus identification”; 2.0 to 2.299 represents “secure genus identification and probable species identification”; and 2.3 to 3.0 indicates a “highly probable species identification”.

### 2.6. Modification of the MSP Identification Method in Biotyper 3 Software

To verify the repeatability of the method, one to nine main spectra from the same host individual were created, depending on the number of AM available, and saved in a MSP database (database 1) (Table 1). However, during these investigations using the protein extraction protocol based on one and 10 AM, slight shifts of the protein spectra of the AM from the same sample on the *x*-axis (mass to charge ratio) were observed. Therefore, the matching of these spectra against one or more already created MSPs from the same sample led to log scores of partially less than 2.0. To solve this problem, the settings of the MSP Identification Method in the Biotyper 3 software were modified as recommended by Bruker (personal communication with the Bruker service department). The aim was that all spectra of AM result in log scores of at least 2.0 compared with the AM-specific MSP library, and that all spectra of *Trichinella* spp. generated by Mayer-Scholl et al. [24] and Karadjian et al. [32] also give log scores of at least 2.0 compared with the *Trichinella* MSP library. Conversely, AM spectra compared with the *Trichinella* MSP library as well as *Trichinella* spp. compared with the AM-specific MSP database should give log scores of less than 1.7. For adaption, single parameters were slightly varied and compared with the resulting score values of the AM and *Trichinella* spp. spectra matched against both the AM- and the *Trichinella*-specific MSP library. Exactly 100 samples of *Trichinella* (*T.*) *spiralis*, *T. pseudospiralis*, *T. britovi*, *T. nativa*, *Hyostrongylus rubidus*, *Trichuris* spp. and an unknown nematode all included in the *Trichinella* database, as well as all AM samples contained in the AM-specific MSP library, were compared with both the AM-specific database and the *Trichinella* MSP library.

Finally, the following settings of the MSP Identification Method were modified: desired mass tolerance of the adjusted spectrum–400 ppm, furthermore accepted mass tolerance of a peak–800 ppm and parameter of the intensity correction function–zero. The remaining parameters (frequency threshold for spectra adjusting, frequency threshold for score calculation and max. mass error of the raw spectrum) were not changed.

### 2.7. Cluster Analysis 

**Figure 1:**
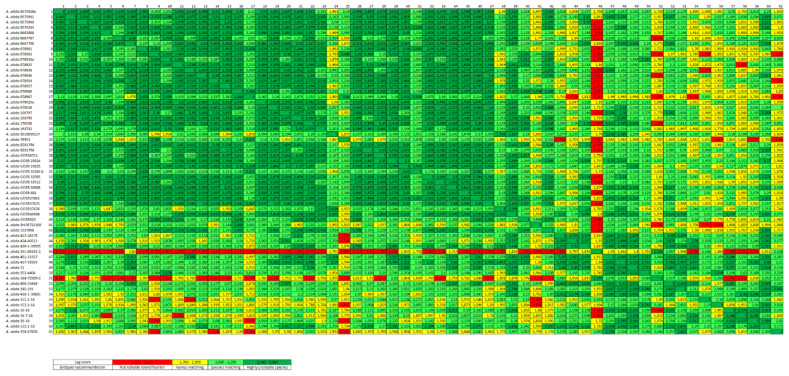
Heat map of 61 *A. alata* main spectra profiles (MSP) based on log scores. Each MSP represents one host individual. The presented figure is sorted by host species, country and region of origin. Nos 1 to 54 are wild boars, nos 55 and 56 are common frogs, nos 57 to 60 are water frogs and no. 61 is a lynx. Further information about the *A. alata* samples are listed in Table 1.

**Figure 2:**
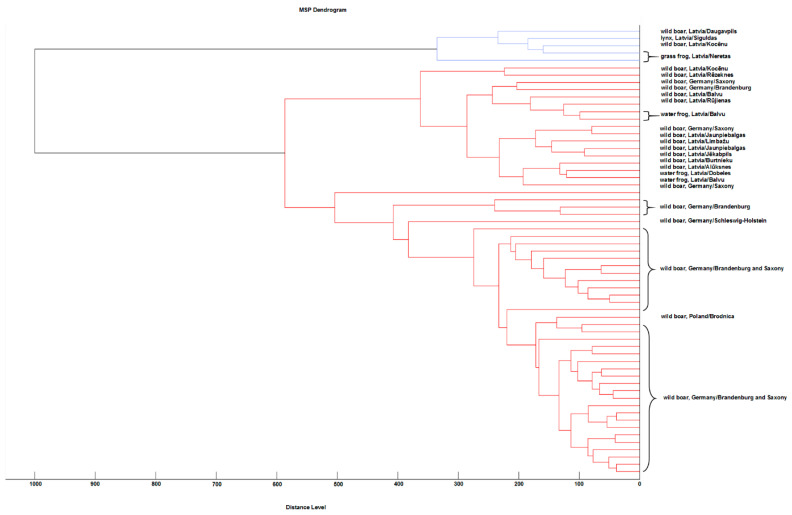
MSP dendrogram cluster analysis of 61 *A. alata* main spectra profiles representing one host individual each.

All of the main spectra were compared using MALDI Biotyper 3 software, and their log score values were converted into a cross table in an Excel spreadsheet using a Biotyper Conversion program created by Holger Brendebach (Department of Biological Safety, Federal Institute for Risk Assessment, Berlin, Germany). Based on this cross table, a heat map of the MSPs was created displaying the described cutoff values with different colors (Figure 1). An MSP dendrogram cluster analysis was performed with a correlation distance measurement and single linkage using MALDI Biotyper 3 software (Figure 2). In the MSP dendrogram, a distance level of zero indicates complete similarity and 1000 means complete dissimilarity (Figure 2).

### 2.8. Validation of the Developed Protocol Based on 10 AM

To guarantee the reproducibility of our method, the 10 AM protocol was tested in two different laboratories: the BIOR in Riga, Latvia and the BfR in Berlin, Germany. For validation, both laboratories tested seven samples (nos 1–7) containing 10 AM each. AM sample nos 1–3 came from the same wild boar from Saxony, Germany; AM samples nos 4–7 were from three different wild boars from Latvia and sample nos 4 and 5 came from the same wild boar. All wild boars from Germany and Latvia had already been included in the AM-specific database.

## 3. Results

### 3.1. Origin of the Samples

In total, 61 AM samples were identified morphologically as *A. alata* using the stereomicroscope. For all samples, these results were confirmed by the *Alaria* spp.-specific PCR [22].

The 61 AM positive muscle samples came from naturally AM infected wild boars (*n* = 54), European water frogs (*n* = 4), common frogs (*n* = 2) and a Eurasian lynx (*n* = 1). 41 wild boars originated from Germany (Brandenburg (25/41), Saxony (15/41) and Schleswig-Holstein (1/41)), one wild boar came from Poland (Brodnica). 19 samples originated from different regions in Latvia and were isolated from wild boars (12/19), European water frogs (4/19), common frogs (2/19) and a Eurasian lynx (1/19) (Table 1).

### 3.2. Molecular Analysis of the Samples

From the 61 *Alaria*-specific PCR positive samples, 20 were further confirmed as *A. alata* by 18S PCR [3] followed by sequencing. In addition, the three samples containing *Opisthioglyphe ranae* larvae were examined by 18S PCR [3] and identified as such (99% identity each).

Interestingly, the 18S sequencing protocol by Karadjian et al. [3] described for identification of nematodes showed complete correlation for the detection of trematodes. However, in all examinations, the fragment length of the PCR products from trematodes was about 750 bp and therefore 100 bp larger than the fragment length of the nematodes described by Karadjian et al. [3] (650 bp).

### 3.3. Creation of Main Spectra Profiles (MSPs)

From all 61 AM samples, a total of 148 main spectra were initially generated using this newly developed MALDI-TOF technique (database 1) (Table 1). After the modification of the MSP Identification Method, 61 of the original 148 main spectra remained. These 61 MSPs represent one host individual each and were included in the newly established AM-specific reference spectra database (database 2).

### 3.4. Cluster Analysis of the Created MSPs

For a cluster analysis of these AM main spectra, a heat map was created where two clusters with different regional origins of AM were observed (Figure 1). The large German cluster contained all wild boar spectra from Germany (nos 1–40) and Poland (no. 42), which showed mainly log scores of at least 2.0. Within this cluster, three main spectra from wild boars from Brandenburg (no. 24 and no. 25) and Saxony (no. 38) demonstrated slight differences (some with log scores between 1.7 to 1.9) (Table 1, Figure 1). In addition, the main spectrum from the German federal state of Schleswig-Holstein (no. 41) presented many score values of 1.7 to 1.9 when compared with the rest of the German cluster, and therefore clearly differed from this cluster. The small Latvian cluster was formed by all the Latvian main spectra from the four different host species: wild boar, common frog, European water frog and Eurasian lynx (nos 43–61). However, two wild boar spectra (no. 46 and no. 51) showed major differences (log scores of 0.7 to 1.9) and were therefore excluded from this cluster. Apart from these two main spectra (no. 46 and no. 51), most wild boar spectra from Latvia were approximately in agreement with the results of the German cluster (nos 43, 45, 47, 48, 49, 50, 52 and 53) or showed only slight variations (no. 44 and no. 54) (Figure 1).

The results described in the heat map are nearly mirrored in the MSP dendrogram depicted in Figure 2. Here, the two clusters can be seen more prominently. The small cluster contained five Latvian spectra from wild boars (no. 46 and no. 51), common frogs (no. 55 and no. 56) and the Eurasian lynx (no. 61) (Figure 2). The big cluster contained two subclusters including 17 (cluster 1), respectively 39 (cluster 2) main spectra. In difference to Figure 1, the first subcluster included both Latvian (wild boars (10), European water frogs (4)) and German (wild boars (3), nos 24, 38 and 39) main spectra, while the second subcluster was formed only by wild boar main spectra from Germany (Saxony, Brandenburg and Schleswig-Holstein) (38) and Poland (Brodnica) (1) (Figure 2).

### 3.5. Sensitivity, Reproducibility and Repeatability of this MALDI-TOF Technique

Further, the three *Opisthioglyphe ranae* samples tested negative for AM using MALDI-TOF MS (log scores less than 1.7), demonstrating that this method also allows a reliable differentiation between trematode species.

The reproducibility of the protocol based on 10 AM was shown as six of seven samples tested in the two separate laboratories showed log score values in the range of 2.2–2.5, and one sample gave values from 1.8 to 2.4.

The repeatability of the one AM protocol was demonstrated by the BfR as 36 of 38 AM showed log scores of 2.0 and more in all three single spectra. Two AM each gave two single spectra with score values of at least 2.0; one spectrum each showed genus matching (log scores of 1876, respectively 1966).

## 4. Discussion

### 4.1. Background and Context to Previous Studies

Due to the high frequency of incidental AM findings in wild boar carcasses and the fact that there is no mandatory AM testing of wild boars to date, an exposure of humans to *A. alata* via the consumption of wild boar meat cannot be excluded.

The presence of AM in second intermediate and paratenic hosts previously reported in several European countries were all based on the morphological identification of AM and/or molecular methods [4,6,7,10,11,12,13,18,19,20]. Furthermore, DNA extraction protocols have been established and several different primer pairs developed to examine the genetic diversity of *A. alata* [22,23,38,39]. By contrast, this study presents MALDI-TOF MS as a rapid, cost-efficient technique to be used as a standard tool and a future trend for the identification of *A. alata*.

To our knowledge, the only other study using MALDI-TOF MS for the identification of *A. alata* is presented by Huguenin et al. [31]. In contrast to our study, the authors showed low intra-species heterogeneity when visualizing *A. alata* cercariae from various snail species from France in a MSP dendrogram (distance level < 100). A similar protein extraction protocol was implemented which only differed in the addition order of the same reagents [31]. However, the protein spectra of *A. alata* cercariae generated in this study did not match with the protein spectra of AM created in our study. This may be due to the slightly differing protein extraction protocols or the different host species. Nevertheless, the varying protein patterns are most likely associated with the different developmental stages of *A. alata* (cercariae vs. mesocercariae), which may be examined in further studies.

### 4.2. Interpretation of the Cluster Analysis

In the present survey, the cluster information obtained through the analysis of 61 MSPs from different host animals indicated possible variation within the *A. alata* species, with a tentative association with the geographical origin of the host, but not the host species. The AM sample from Poland, which is the neighboring region of East Germany, clusters with the East-German samples. By contrast, the Latvian AM samples originating from North-Eastern Europe formed their own cluster, even if some wild boar spectra and one water frog spectrum also fit in the above-mentioned German/Polish cluster. A total of 12 spectra (11 from the North-Eastern and one from the German/Polish group) did not cluster according to geographical origin. At this point, the possibility of identifying intra-species variability is purely speculative due to the small size of the samples in this study. Nevertheless, Bilska-Zając et al. [23] recently published a manuscript describing intraspecific genetic variability among AM specimens, i.e., 17 different genotypes of AM. However, in this study, a direct association between the genotype of this parasite and the host’s geographical origin was not observed [23].

### 4.3. Future Potential of the MALDI-TOF Technique

Currently, the identification of zoonotic or potentially zoonotic parasitic species other than *Trichinella* spp. is fully dependent on either morphological classification [15] and/or PCR [22] or even PCR followed by sequencing [3,40], making this procedure unsuitable for use in routine laboratories. As the MALDI-TOF technique has been implemented in many routine diagnostic laboratories in past years, a generally available protocol for the analysis of parasitic pathogens and contaminants would be advantageous. The specificity of the MALDI-TOF method is fully sufficient to distinguish between the genus *Trichinella* and *Alaria* (log score values < 1.700, data not shown), and even has the potential to distinguish between *Trichinella* species, or perhaps even between genotypes [24].

In this study, a future trend of MALDI-TOF MS is presented as a standard tool for the identification of *A. alata*, including the capability of differentiation between trematode species. Currently, a protocol is under development to harmonize the protein extraction for both nematodes and trematodes isolated from meat.

## 5. Conclusions

The aim of this study was to develop a standardized MALDI-TOF assay for the rapid and reliable identification of AM in wild boar meat. Protein extraction protocols based on one and 10 AM were established and pre-validated. Furthermore, an AM-specific reference spectra database including 61 MSPs from different host individuals was created.

The long-term objective is to develop a unique protein extraction protocol and to generate a universal database for the identification of several parasites (e.g., *Trichinella* spp., *Toxocara canis/cati*, *Ascaris suum*, *Metastrongylus* spp. and *Uncinaria stenocephala*) isolated from meat.

## Figures and Tables

**Table 1:** List of *Alaria alata* mesocercariae included in this study. The 8th column of this table gives the log score values for comparison of at least two main spectra profiles created from the same host individual.

No.	Sample Number	Year of Sampling	Host Species	Origin of the Host	Host Tissue Used for AM Isolation	Number of MSPs	Log Scores for MSPs from the Same Host
1	*A. alata* 0573928a	2018	wild boar	Germany, Brandenburg	tongue	2	2.474
2	*A. alata* 0573941	2018	wild boar	Germany, Brandenburg	tongue	1	no value
3	*A. alata* 0573944	2018	wild boar	Germany, Brandenburg	tongue	1	no value
4	*A. alata* 0574243	2018	wild boar	Germany, Brandenburg	tongue	2	2.592–2.593
5	*A. alata* 0662088	2019	wild boar	Germany, Brandenburg	foreleg muscles	1	no value
6	*A. alata* 0667797	2019	wild boar	Germany, Brandenburg	tongue	1	no value
7	*A. alata* 0667798	2019	wild boar	Germany, Brandenburg	tongue	3	2.062–2.599
8	*A. alata* 078901	2019	wild boar	Germany, Brandenburg	tongue, abdominal fat tissue	5	2.383–2.660
9	*A. alata* 078902	2019	wild boar	Germany, Brandenburg	tongue, abdominal fat tissue	5	2.316–2.638
10	*A. alata* 078924a	2019	wild boar	Germany, Brandenburg	tongue, abdominal fat tissue	5	2.011–2.562
11	*A. alata* 078925	2019	wild boar	Germany, Brandenburg	tongue, abdominal fat tissue	1	no value
12	*A. alata* 078939	2019	wild boar	Germany, Brandenburg	tongue, abdominal fat tissue	4	2.308–2.550
13	*A. alata* 078946	2019	wild boar	Germany, Brandenburg	tongue, abdominal fat tissue	1	no value
14	*A. alata* 078954	2019	wild boar	Germany, Brandenburg	tongue, abdominal fat tissue	5	2.318–2.623
15	*A. alata* 078957	2019	wild boar	Germany, Brandenburg	tongue, abdominal fat tissue	4	2.127–2.430
16	*A. alata* 078966	2019	wild boar	Germany, Brandenburg	tongue, abdominal fat tissue	1	no value
17	*A. alata* 078987	2019	wild boar	Germany, Brandenburg	tongue, abdominal fat tissue	5	2.341–2.767
18	*A. alata* 079025a	2019	wild boar	Germany, Brandenburg	tongue, abdominal fat tissue	2	2.625–2.632
19	*A. alata* 079028	2019	wild boar	Germany, Brandenburg	tongue, abdominal fat tissue	4	2.337–2.647
20	*A. alata* 106797	2019	wild boar	Germany, Brandenburg	tongue, abdominal fat tissue	5	2.013–2.597
21	*A. alata* 106799	2019	wild boar	Germany, Brandenburg	tongue, abdominal fat tissue	1	no value
22	*A. alata* 170238	2019	wild boar	Germany, Brandenburg	tongue, abdominal fat tissue	1	no value
23	*A. alata* 193732	2019	wild boar	Germany, Brandenburg	tongue, abdominal fat tissue	1	no value
24	*A. alata* 2019030127	2020	wild boar	Germany, Brandenburg	tongue, abdominal fat tissue, diaphragm, chewing muscles	3	2.488–2.681
25	*A. alata* 78953	2019	wild boar	Germany, Brandenburg	tongue, abdominal fat tissue	5	2.108–2.675
26	*A. alata* BZ41794	2019	wild boar	Germany, Saxony	tongue, foreleg muscles	1	no value
27	*A. alata* BZ41796	2019	wild boar	Germany, Saxony	tongue, foreleg muscles	1	no value
28	*A. alata* GOER36711	2018	wild boar	Germany, Saxony	diaphragm, stomach, chewing muscles	1	no value
29	*A. alata* GOER23024	2019	wild boar	Germany, Saxony	diaphragm, stomach, chewing muscles	5	2.012–2.491
30	*A. alata* GOER23025	2019	wild boar	Germany, Saxony	diaphragm, stomach, chewing muscles	5	2.368–2.545
31	*A. alata* GOER32365b	2019	wild boar	Germany, Saxony	diaphragm, stomach, chewing muscles	3	2.492–2.707
32	*A. alata* GOER32505	2018	wild boar	Germany, Saxony	diaphragm, stomach, chewing muscles	1	no value
33	*A. alata* GOER33512	2019	wild boar	Germany, Saxony	diaphragm, stomach, chewing muscles	4	2.468–2.575
34	*A. alata* GOER36908	2019	wild boar	Germany, Saxony	diaphragm, chewing muscles, abdominal fat tissue	5	2.167–2.497
35	*A. alata* GOER481	2018	wild boar	Germany, Saxony	diaphragm, stomach, chewing muscles	1	no value
36	*A. alata* GOER37662	2019	wild boar	Germany, Saxony	diaphragm, stomach, chewing muscles	4	2.455–2.619
37	*A. alata* GOER37673	2020	wild boar	Germany, Saxony	diaphragm, stomach, chewing muscles	4	2.305–2.587
38	*A. alata* GOER37676	2020	wild boar	Germany, Saxony	diaphragm, stomach, chewing muscles	1	no value
39	*A. alata* GOER44948	2020	wild boar	Germany, Saxony	diaphragm, stomach, chewing muscles	3	2.668–2.747
40	*A. alata* GOER925	2020	wild boar	Germany, Saxony	diaphragm, stomach, chewing muscles	5	2.000–2.508
41	*A. alata* SH05721106	2019	wild boar	Germany, Schleswig-Holstein	diaphragm	1	no value
42	*A. alata* 1537008	2019	wild boar	Poland, Brodnica	diaphragm	5	2.175–2.579
43	*A. alata* 422-24178	2020	wild boar	Latvia, Alūksnes	diaphragm	1	no value
44	*A. alata* 424-40513	2020	wild boar	Latvia, Balvu	diaphragm	1	no value
45	*A. alata* 420-1-39905	2020	wild boar	Latvia, Burtnieku	diaphragm	2	2.428–2.444
46	*A. alata* 231-88191-2	2018	wild boar	Latvia, Daugavpils	diaphragm, tongue	1	no value
47	*A. alata* 401-31537	2020	wild boar	Latvia, Jaunpiebalgas	diaphragm	1	no value
48	*A. alata* 417-39359	2020	wild boar	Latvia, Jaunpiebalgas	diaphragm	1	no value
49	*A. alata* 11	2020	wild boar	Latvia, Jēkabpils	diaphragm	2	2.520–2.523
50	*A. alata* 252-4404	2019	wild boar	Latvia, Kocēnu	diaphragm, tongue	1	no value
51	*A. alata* 184-72509-2	2018	wild boar	Latvia, Kocēnu	diaphragm	1	no value
52	*A. alata* 408-33448	2020	wild boar	Riga, Limbažu	diaphragm	1	no value
53	A. alata 245-191	2019	wild boar	Latvia, Rēzeknes	diaphragm, tongue	1	no value
54	*A. alata* 418-1-39881	2020	wild boar	Latvia, Rūjienas	diaphragm	9	2.181–2.745
55	*A. alata* 151.3-18	2018	common frog	Latvia, Neretas	serous coat of the internal organs	1	no value
56	*A. alata* 152.1-18	2018	common frog	Latvia, Neretas	head	1	no value
57	*A. alata* 10-18	2018	water frog	Latvia, Balvu	serous coat of the internal organs	1	no value
58	*A. alata* 16.7-18	2018	water frog	Latvia, Balvu	inner organs	1	no value
59	*A. alata* 20-18	2018	water frog	Latvia, Balvu	serous coat of the internal organs	1	no value
60	*A. alata* 132.1-18	2018	water frog	Latvia, Dobeles	head	1	no value
61	*A. alata* 238-87828	2018	lynx	Latvia, Siguldas	tongue, different muscles	1	no value

*A*., *Alaria*; AM, *A. alata* mesocercariae; MSP, main spectrum profile. Note: Sample nos 1–25 came from the prevalence study in Brandenburg, Germany, sample nos 26–42 originating from official *Trichinella* testing were collected by the BfR, Germany, and sample nos 43–61 were gathered by BIOR, Latvia, for research purposes.

## References

[1] European Commission (2015). Commission Implementing Regulation (EU) 2015/1375 of 10 August 2015 Laying down Specific Rules on Official Controls for Trichinella in Meat. Off. J. Eur. Union.

[2] European Commission (2020). Commission Implementing Regulation (EU) 2020/1478 of 14 October 2020 amending Implementing Regulation (EU) 2015/1375 as regards sampling, the reference method for detection and import conditions related to *Trichinella* control C.I.R. Off. J. Eur. Union.

[3] Karadjian G., Kaestner C., Laboutière L., Adicéam E., Wagner T., Johne A., Thomas M., Polack B., Mayer-Scholl A., Vallée I. (2020). A two-step morphology-PCR strategy for the identification of nematode larvae recovered from muscles after artificial digestion at meat inspection. Parasitol. Res..

[4] Riehn K., Hamedy A., Grosse K., Wüste T., Lücker E. (2012). *Alaria alata* in wild boars (*Sus scrofa*, Linnaeus, 1758) in the eastern parts of Germany. Parasitol. Res..

[5] Sailer A., Glawischnig W., Irschik I., Lücker E., Riehn K., Paulsen P. (2012). Findings of *Alaria alata* mesocercariae in wild boar in Austria: Current knowledge, identification of risk factors and discussion of risk management options. Wien. Tierärztliche Mon..

[6] Paulsen P., Forejtek P., Hutarova Z., Vodnansky M. (2013). *Alaria alata* mesocercariae in wild boar (*Sus scrofa*, Linnaeus, 1758) in south regions of the Czech Republic. Vet. Parasitol..

[7] Portier J., Vallée I., Lacour S.A., Martin-Schaller R., Ferté H., Durand B. (2014). Increasing circulation of *Alaria alata* mesocercaria in wild boar populations of the Rhine valley, France, 2007–2011. Vet. Parasitol..

[8] Riehn K., Lalkovski N., Hamedy A., Lücker E. (2014). First detection of *Alaria alata* mesocercariae in wild boars (*Sus scrofa* Linnaeus, 1758) from Bulgaria. J. Helminthol..

[9] Maleševic M., Smulders F.J.M., Petrovic J., Mirceta J., Paulsen P. (2016). *Alaria alata* mesocercariae in wild boars (*Sus scrofa*) in northern Serbia after the flood disaster of 2014. Wien. Tierarztl. Mon..

[10] Ozoliņa Z., Mateusa M., Šuksta L., Liepiņa L., Deksne G. (2020). The wild boar (*Sus scrofa*, Linnaeus1758) as an important reservoir host for *Alaria alata* in the Baltic region and potential risk of infection in humans. Vet. Parasitol. Reg. Stud. Rep..

[11] Kästner C., Bier N.S., Mayer-Scholl A., Nöckler K., Richter M.H., Johne A. (2021). Prevalence of *Alaria alata* mesocercariae in wild boars from Brandenburg, Germany. Parasitol. Res..

[12] Strokowska N., Nowicki M., Klich D., Bełkot Z., Wiśniewski J., Didkowska A., Chyla P., Anusz K. (2020). The occurrence of *Alaria alata* mesocercariae in wild boars (*Sus scrofa*) in north-eastern Poland. Int. J. Parasitol. Parasites Wildl..

[13] Gazzonis A.L., Villa L., Riehn K., Hamedy A., Minazzi S., Olivieri E., Zanzani S.A., Manfredi M.T. (2018). Occurrence of selected zoonotic food-borne parasites and first molecular identification of *Alaria alata* in wild boars (*Sus scrofa*) in Italy. Parasitol. Res..

[14] Odening K. (1961). Der “Dunckersche Muskelegel” kann experimentell auf Affen übertragen werden. Mon. Für Veterinärmedizin.

[15] Möhl K., Große K., Hamedy A., Wüste T., Kabelitz P., Lücker E. (2009). Biology of *Alaria* spp. and human exposition risk to *Alaria* mesocercariae-a review. Parasitol. Res..

[16] Gottstein B. (2013). Einstufung von Organismen. Modul 3: Parasiten. Stand Januar 2013. Herausgegeben vom Bundesamt für Umwelt BAFU, Bundesamt für Gesundheit. http://www.bafu.admin.ch/uv-1114-d.

[17] (2020). Federal Ministry of Labour and Social Affairs, Technische Regeln für Biologische Arbeitsstoffe (TRBA) 464 “Einstufung von Parasiten in Risikogruppen”, Ausgabe Juli 2013, 1. Änderung vom 10.11.2020, GMBI Nr. 45. https://www.baua.de/DE/Angebote/Rechtstexte-und-Technische-Regeln/Regelwerk/TRBA/pdf/TRBA464.pdf?__blob=publicationFile&v=3.

[18] (2016). BVL, Zoonosen-Monitoring 2015. Gemeinsamer Bericht des Bundes und der Länder. Berichte zur Lebensmittelsicherheit, BVL-Report 11.2: 30-31. https://www.bvl.bund.de/SharedDocs/Downloads/01_Lebensmittel/04_Zoonosen_Monitoring/Zoonosen_Monitoring_Bericht_2015.pdf%3F__blob%3DpublicationFile%26v%3D6.

[19] Gavrilović P., Pavlović I., Todorović I. (2019). *Alaria alata* mesocercariae in domestic pigs and wild boars in South Banat, northern Serbia. Comp. Immunol. Microbiol. Infect. Dis..

[20] Paulsen P., Ehebruster J., Irschik I., Lücker E., Riehn K., Winkelmayer R., Smulders F.J.M. (2012). Findings of *Alaria alata* mesocercariae in wild boars (*Sus scrofa*) in eastern Austria. Eur. J. Wildl. Res..

[21] Riehn K., Hamedy A., Große K., Zeitler L., Lücker E. (2010). A novel detection method for *Alaria alata* mesocercariae in meat. Parasitol. Res..

[22] Riehn K., Hamedy A., Alter T., Lücker E. (2011). Development of a PCR approach for differentiation of *Alaria* spp. mesocercariae. Parasitol. Res..

[23] Bilska-Zając E., Marucci G., Piróg-Komorowska A., Cichocka M., Różycki M., Karamon J., Sroka J., Bełcik A., Mizak I., Cencek T. (2020). Occurrence of *Alaria alata* in wild boars (*Sus scrofa*) in Poland and detection of genetic variability between isolates. Parasitol. Res..

[24] Mayer-Scholl A., Murugaiyan J., Neumann J., Bahn P., Reckinger S., Nöckler K. (2016). Rapid Identification of the Foodborne Pathogen *Trichinella* spp. by Matrix-Assisted Laser Desorption/Ionization Mass Spectrometry. PLoS ONE.

[25] Wieser A., Schneider L., Jung J., Schubert S. (2012). MALDI-TOF MS in microbiological diagnostics-identification of microorganisms and beyond (mini review). Appl. Microbiol. Biotechnol..

[26] Shannon S., Kronemann D., Patel R., Schuetz A.N. (2018). Routine use of MALDI-TOF MS for anaerobic bacterial identification in clinical microbiology. Anaerobe.

[27] Croxatto A., Prod’hom G., Greub G. (2012). Applications of MALDI-TOF mass spectrometry in clinical diagnostic microbiology. FEMS Microbiol. Rev..

[28] Bredtmann C.M., Krücken J., Murugaiyan J., Kuzmina T., von Samson-Himmelstjerna G. (2017). Nematode species identification—current status, challenges and future perspectives for Cyathostomins. Front. Cell. Infect. Microbiol..

[29] Marzano V., Pane S., Foglietta G., Levi Mortera S., Vernocchi P., Onetti Muda A., Putignani L. (2020). Mass Spectrometry Based-Proteomic Analysis of *Anisakis* spp.: A Preliminary Study towards a New Diagnostic Tool. Genes.

[30] Sy I., Margardt L., Ngbede E.O., Adah M.I., Yusuf S.T., Keiser J., Utzinger J., Poppert S., Becker S.L. (2021). Identification of Adult *Fasciola* spp. Using Matrix-Assisted Laser/Desorption Ionization Time-of-Flight (MALDI-TOF) Mass Spectrometry. Microorganisms.

[31] Huguenin A., Depaquit J., Villena I., Ferté H. (2019). MALDI-TOF mass spectrometry: A new tool for rapid identification of cercariae (Trematoda, Digenea). Parasite.

[32] Karadjian G., Bilska-Zając E., Bahn P., Py J.-S., Johne A., Gassilloud B., Różycki M., Cencek T., Mayer-Scholl A., Vallée I. (2020). Species identification of *Trichinella* originated from various host and different geographical location by MALDI-TOF. Exp. Parasitol..

[33] Ozoliņa Z., Bagrade G., Deksne G. (2020). First confirmed case of *Alaria alata* mesocercaria in Eurasian lynx (Lynx lynx) hunted in Latvia. Parasitol. Res..

[34] Ozoliņa Z., Deksne G., Pupins M., Gravele E., Gavarane I., Kirjušina M. (2020). *Alaria alata* mesocercariae prevalence and predilection sites in amphibians in Latvia. Parasitol. Res..

[35] Guillen J. (2012). FELASA guidelines and recommendations. J. Am. Assoc. Lab. Anim. Sci..

[36] Justine J.-L., Briand M.J., Bray R.A. (2012). A quick and simple method, usable in the field, for collecting parasites in suitable condition for both morphological and molecular studies. Parasitol. Res..

[37] Khalil M.I., El-Shahawy I.S., Abdelkader H.S. (2014). Studies on some fish parasites of public health importance in the southern area of Saudi Arabia. Rev. Bras. Parasitol. Veterinária.

[38] Olson P.D., Cribb T., Tkach V., Bray R., Littlewood D. (2003). Phylogeny and classification of the Digenea (Platyhelminthes: Trematoda). Int. J. Parasitol..

[39] Portier J., Jouet D., Vallee I., Ferté H. (2012). Detection of *Planorbis planorbis* and *Anisus vortex* as first intermediate hosts of *Alaria alata* (Goeze, 1792) in natural conditions in France: Molecular evidence. Vet. Parasitol..

[40] Marucci G., Interisano M.M., La Rosa G., Pozio E. (2013). Molecular identification of nematode larvae different from those of the *Trichinella* genus detected by muscle digestion. Vet. Parasitol..

